# Monitoring Bacterial Burden, Inflammation and Bone Damage Longitudinally Using Optical and μCT Imaging in an Orthopaedic Implant Infection in Mice

**DOI:** 10.1371/journal.pone.0047397

**Published:** 2012-10-17

**Authors:** Jared A. Niska, Jeffrey A. Meganck, Jonathan R. Pribaz, Jonathan H. Shahbazian, Ed Lim, Ning Zhang, Brad W. Rice, Ali Akin, Romela Irene Ramos, Nicholas M. Bernthal, Kevin P. Francis, Lloyd S. Miller

**Affiliations:** 1 Orthopaedic Hospital Research Center, Orthopaedic Hospital Department of Orthopaedic Surgery, David Geffen School of Medicine at University of California Los Angeles (UCLA), Los Angeles, California, United States of America; 2 Caliper – a PerkinElmer Company, Alameda, California, United States of America; 3 Department of Dermatology, Johns Hopkins University School of Medicine, Baltimore, Maryland, United States of America; 4 Novartis Institutes for BioMedical Research, Inc., Emeryville, California, United States of America; University of São Paulo, Brazil

## Abstract

**Background:**

Recent advances in non-invasive optical, radiographic and μCT imaging provide an opportunity to monitor biological processes longitudinally in an anatomical context. One particularly relevant application for combining these modalities is to study orthopaedic implant infections. These infections are characterized by the formation of persistent bacterial biofilms on the implanted materials, causing inflammation, periprosthetic osteolysis, osteomyelitis, and bone damage, resulting in implant loosening and failure.

**Methodology/Principal Findings:**

An orthopaedic implant infection model was used in which a titanium Kirshner-wire was surgically placed in femurs of LysEGFP mice, which possess EGFP-fluorescent neutrophils, and a bioluminescent *S. aureus* strain (Xen29; 1×10^3^ CFUs) was inoculated in the knee joint before closure. *In vivo* bioluminescent, fluorescent, X-ray and μCT imaging were performed on various postoperative days. The bacterial bioluminescent signals of the *S. aureus*-infected mice peaked on day 19, before decreasing to a basal level of light, which remained measurable for the entire 48 day experiment. Neutrophil EGFP-fluorescent signals of the *S. aureus*-infected mice were statistically greater than uninfected mice on days 2 and 5, but afterwards the signals for both groups approached background levels of detection. To visualize the three-dimensional location of the bacterial infection and neutrophil infiltration, a diffuse optical tomography reconstruction algorithm was used to co-register the bioluminescent and fluorescent signals with μCT images. To quantify the anatomical bone changes on the μCT images, the outer bone volume of the distal femurs were measured using a semi-automated contour based segmentation process. The outer bone volume increased through day 48, indicating that bone damage continued during the implant infection.

**Conclusions/Significance:**

Bioluminescent and fluorescent optical imaging was combined with X-ray and μCT imaging to provide noninvasive and longitudinal measurements of the dynamic changes in bacterial burden, neutrophil recruitment and bone damage in a mouse orthopaedic implant infection model.

## Introduction

Over the past decade, whole animal *in vivo* optical imaging, such as noninvasive bioluminescent and fluorescent imaging, has emerged as a powerful technique that provides the ability to monitor different biological processes dynamically and longitudinally over time in a living animal [1]–[4]. This technology has provided key conceptual advances in disease and therapy in many different fields of biomedical research, including immunology and infectious diseases [1]–[4]. *In vivo* bioluminescent imaging involves the detection of light produced by luciferase enzymes [1]–[3]. Regarding infectious diseases, many different bacterial species have been genetically-engineered to express luciferase enzymes from natural light producing organisms [5], [6]. These transformed bioluminescent bacterial strains can be used to track both the location and the burden of the microbe *in vivo* [5], [6]. In particular, the lux operon from *Photorhabdus luminescens* has commonly been used for this purpose, since it encodes all of the proteins necessary for bioluminescence [5], [6]. Thus, bacterial strains expressing modified versions of this lux operon do not require an exogenous substrate for light production and only live, actively metabolizing bacteria will emit light [5], [6]. *In vivo* fluorescent imaging involves excitation of a fluorescent molecule by an external light source and detection of an emitted wavelength of light [3], [4]. Fluorescent imaging can be used in combination with bioluminescent imaging to allow independent information to be gathered sequentially, such as the accumulation of fluorescently-labeled immune cells to the site of a bacterial infection [7]–[9]. Bioluminescent and fluorescent signals are typically detected in anesthetized mice placed in an imaging chamber using highly-sensitive charged-coupled device (CCD) cameras [1]–[4]. The optical signals are then automatically superimposed on digital photographic images of the mice to determine the signal intensity and two-dimensional (2D) anatomical location [1]–[4].

In our prior work, we demonstrated that a particularly useful application of *in vivo* bioluminescence and fluorescence optical imaging is to study orthopaedic implant infections [10]–[13]. Clinically, these infections are characterized by the formation of bacterial biofilms on the implanted materials that results in a persistent bacterial infection [14]–[16]. The ongoing infection and inflammatory response leads to periprosthetic osteolysis, osteomyelitis, bone damage and ultimately implant loosening and failure [17]–[19]. These infections represent a major complication in orthopaedic surgery because the treatment involves extensive medical and surgical care, including multiple reoperations for surgical debridement and to remove the infected implanted materials, prolonged systemic antibiotic therapy and extended disability and rehabilitation, which contribute to worse clinical outcomes [17], [18], [20]. Furthermore, the medical costs of treating orthopaedic implant infections are enormous [19]. For example, the additional inpatient hospital costs for infected total knee and hip replacements average ∼$30,000 per patient (not including outpatient costs or costs of extended disability and lost wages) or an annual national healthcare burden that is approaching $1 billion in the United States [21].

To use *in vivo* optical imaging techniques to study these infections, we developed a mouse model of orthopaedic implant infection which involves the surgical placement of a metal Kirshner-wire (K-wire) implant into the right femurs of mice followed by the inoculation of a bioluminescent *Staphylococcus aureus* strain into the joint space before closure [10]–[13]. *S. aureus* was chosen as a representative bacterial species because both methicillin-sensitive *S. aureus* (MSSA) and methicillin-resistant *S. aureus* (MRSA) are clinically relevant causes of orthopaedic implant infections [22]. *In vivo* bioluminescent bacterial signals were measured longitudinally over time using the IVIS Lumina II® imaging system (Caliper – a PerkinElmer Company, Alameda, CA). In addition, the numbers of neutrophils at the site of infection correlates with the presence of an orthopaedic implant infection [23], [24], Therefore, LysEGFP mice, a genetically engineered mouse strain that possesses EGFP-fluorescent neutrophils [25], were employed in this model to quantify neutrophil infiltration to the site of infection using sequential *in vivo* fluorescence imaging using the same imaging system [11], [13]. This mouse model was successfully utilized to determine the efficacy of local and systemic therapeutic strategies [10], [13] and to investigate protective immune responses against these implant infections [12]. However, a limitation of this model was the inability to assess the consequences of the infection and inflammation, especially on the pathologic changes that occur in the bone, which result in the periprosthetic osteolysis, implant loosening and clinical failure.

Recently, advances in *in vivo* imaging technology have provided the opportunity to combine *in vivo* optical imaging with structural imaging modalities such as X-ray, computed tomography (CT), and magnetic resonance imaging (MRI) to monitor biological processes in a three-dimensional (3D) anatomical context [5], [6]. These multimodality imaging technologies are becoming increasingly accessible as commercially-available imaging systems have been developed to observe and quantify the spatial and temporal distribution of optical signals from a living animal in 3D [6], [26]. In addition, this multimodality optical and anatomical imaging may be particularly effective in studying biological processes that involve dynamic changes in bone [26]–[28]. Therefore, in this study, we attempted to use multimodality optical and anatomical imaging to better evaluate noninvasively and longitudinally the bacterial burden and neutrophilic inflammation in the context of the pathologic changes that occur in bone in our mouse model of orthopaedic implant infection.

## Materials and Methods

### Ethics statement

All animals were handled in strict accordance with good animal practice as defined in the federal regulations as set forth in the Animal Welfare Act (AWA), the 1996 Guide for the Care and Use of Laboratory Animals, PHS Policy for the Humane Care and Use of Laboratory Animals, as well as UCLA's policies and procedures as set forth in the UCLA Animal Care and Use Training Manual, and all animal work was approved by the UCLA Chancellor's Animal Research Committee (ARC#: 2008-112).

### *Staphylococcus aureus* bioluminescent strain

The bioluminescent *Staphylococcus aureus* strain, Xen29, used in this study was derived from the pleural fluid isolate ATCC 12600 [29]. Xen29 possesses a stable chromosomally-integrated modified *luxABCDE* operon from the bacterial insect pathogen, *Photorhabdus luminescens*. These bacteria constitutively emit blue-green bioluminescent light and only live and metabolically active bacteria will emit light [29]. This strain maintains the bioluminescent construct in all progeny without selection [29].

### Preparation of *S. aureus* for inoculation into the joint space

Xen29 bacteria were streaked onto tryptic soy agar plates (tryptic soy broth [TSB] containing 1.5% bacto agar [BD Biosciences, Franklin Lakes, NJ]) and grown at 37°C overnight [11]. Single bacterial colonies were cultured in TSB and grown overnight at 37°C in a shaking incubator (240 rpm) (MaxQ 4450; Thermo Fisher Scientific, Waltham, MA). Mid-logarithmic phase bacteria were obtained after a 2 hour subculture of a 1/50 dilution of the overnight culture. Bacteria were pelleted, resuspended and washed 3 times in PBS. Bacterial inocula (1×10^3^ colony forming units [CFUs] in 2 µl PBS) were estimated by measuring the absorbance at 600 nm (Biomate 3; Thermo Fisher Scientific). CFUs were verified after overnight culture on plates.

### Mice

Twelve-week old male LysEGFP mice, a genetically engineered mouse line that possesses green-fluorescent myeloid cells (mostly neutrophils) due to a knockin of enhanced green fluorescence protein (EGFP) into the lysozyme M gene, were used [25].

### Mouse surgical procedures

All procedures were approved by the UCLA Animal Research Committee. Mice were anesthetized via inhalation isoflurane (2%). To model an orthopaedic implant infection, a medical-grade titanium Kirschner-wire (K-wire) was surgically placed into the right distal femur of mice as previously described [11]. Briefly, a skin incision was made over the right knee and the distal right femur was accessed through a medial parapatellar arthrotomy with lateral displacement of the quadriceps-patellar complex. After locating the femoral intercondylar notch, the femoral intramedullary canal was manually reamed with a 25 gauge for entry into the canal and further reamed with a 23 gauge needle. A titanium K-wire (0.8 mm in diameter; Synthes, Inc., West Chester, PA) was surgically placed in a retrograde fashion and cut with 1 mm protruding into the joint space. After cutting, the length of the K-wires within the mouse femurs ranged from 7 to 10 mm in length. An inoculum of Xen29 (1×10^3^ CFUs in 2 µl PBS) or no bacteria (2 µl sterile PBS) (n = 8 mice per group) was pipetted into the joint space containing the cut end of the implant using a micropipette. The quadriceps-patellar complex was reduced to its anatomic position and the surgical site was closed using Vicryl 5-0 sutures. Sustained-release buprenorphine (2.5 mg/kg) (ZooPharm, WY) was administered at the time of surgery and every 3 days postoperatively.

### Two-dimensional optical/X-ray image acquisition and analysis of bacterial burden and neutrophil infiltration (*in vivo* bioluminescence, fluorescence and X-ray imaging)

LysEGFP were anesthetized with inhalation isoflurane (2%), the right hind limb was shaved and *in vivo* Xen29 bioluminescent signals were acquired with a 5 minute imaging time, a 13 cm field of view (FOV), bin 4 and f1 and *in vivo* EGFP-neutrophil fluorescence signals were sequentially acquired with a 465 nm excitation filter and a 520 nm emission filter using bin 8 and f2 using the IVIS Spectrum® imaging system (Caliper – a PerkinElmer Company, Alameda, California). In addition, to view the optical signals in the context of 2D X-ray images of the post-surgical legs, *in vivo* Xen29 bioluminescence signals were acquired with a 5 minute imaging time, a 5 cm FOV, bin 4 and f1, *in vivo* EGFP-neutrophil fluorescence signals were acquired with a 465 nm excitation filter and an EGFP emission filter (515–575 nm) using bin 4 and f2 and X-ray imaging was performed using the IVIS Lumina XR® imaging system (Caliper). Mice were imaged on days 2, 5, 14, 19, 28, 33 and 48. Data are presented on color scale overlaid on a grayscale photograph of the mice or X-ray image of the mice and *in vivo* bioluminescent signals were quantified as total flux (photons per second (s)) and *in vivo* fluorescent signals were quantified as total radiant efficiency ([photons/s]/[µW/cm^2^]) within a circular region of interest (ROI) using Living Image® software (Caliper).

### Three-dimensional optical image acquisition, formation and computed tomography (CT) scan co-registration

To visualize the *in vivo* Xen29 bacterial bioluminescence signals and the *in vivo* EGFP-neutrophil fluorescence signals in a 3D anatomical context, selected LysEGFP mice were placed into a mouse imaging shuttle chamber that could be placed in both the IVIS Spectrum® imaging system and the Quantum FX® *in vivo* μCT system (Caliper). In the IVIS Spectrum®, a series of images were acquired for the 560, 580 and 600 nm emission filters with bin 16 and a 5 minute imaging time per filter. A structured light image was used to generate the surface topography. Optical data were mapped onto this surface for use in a diffuse optical tomography reconstruction algorithm that used a non-negative least squares optimization to reconstruct the 3D optical image with a 1.25 mm voxel size [30]. To visualize the anatomical location of the 3D optical signals, the same mice in the mouse imaging shuttle chamber were sequentially scanned in the Quantum FX® *in vivo* μCT system. The reconstructed μCT image included a fiducial that was used for automatic registration with the 3D optical images generated in the IVIS Spectrum®. Due to slight inaccuracies in the surface topography map created by the structured light surface and black fur, the image registration was then manually adjusted to better align the skin surface on the μCT image with this surface topography map. Finally, a threshold was applied to the μCT image to enable visualization of the bones and the K-wire implants with the 3D optical signals.

### μCT image acquisition

Since image artifacts from the metal K-wires could theoretically cause artifacts in the reconstructed μCT images, 0.8 mm titanium K-wires were used instead of 0.6 mm stainless steel K-wires based on pilot studies showing fewer artifacts with titanium (data not shown). Thus, titanium K-wires were used in all experiments in this study. In addition, low magnification was used to keep the radiation dose low enough for longitudinal studies. Images acquired for the purpose of co-registration used a 60 mm FOV with a 118 µm voxel size and an approximate dose of 26 mGy per scan. To improve the spatial resolution for quantification, images were also obtained for longitudinal analysis using a 30 mm FOV and 59 µm voxel size and an approximate dose of 18 mGy per scan.

### μCT image visualization and analysis

Reconstructed μCT images were initially visualized in 3D to get a sense of changes in bone morphology that occurred as a result of the implant infection. To do this, a threshold limited 3D rendering was created to visualize the bone damage. In addition, clipping planes were applied to limit this 3D rendering to a thick cross-sectional section of the distal femur. An image analysis approach was designed specifically to evaluate the outer bone volume of the distal femur to measure the anatomical change. Briefly, the femoral length in both limbs was measured and the number of slices corresponding to 25% of the femoral length was calculated. The right femur was then reoriented to align the long axis of the femur with a Cartesian axis of the image. Using a semi-automated approach, contours were created no more than every 5^th^ slice for the distal 25% of the femur. This semi-automated process prevented image artifacts from the titanium K-wire from biasing the segmentation. A 3D region of interest was created by propagating between these contours and volume of this ROI was measured (Analyze 10.0, AnalyzeDirect Inc., Overland Park, KS). To visualize these results, the reoriented image was loaded and a 3D rendering was generated from the anterolateral perspective using a fixed threshold range across all of the time points. The outcome measure of interest was a change in the distal femoral outer bone volume over time. Since each bone had a different outer bone volume at the start of the study, outer bone volumes from later time points (i.e., days 5, 14, 28 and 48) were normalized to the earliest imaged time point (i.e., day 2) using the formula: Δ Volume (%) = ([Volume_(day X)_−Volume_(day 2)_]/[Volume_(day 2)_])×100 where the variable “X” represented the time point of interest (i.e., days 5, 14, 28 and 48).

### Quantification of bacteria adherent to the implants and in the joint tissue

On day 48, mice were euthanized and the implants and joint tissue were harvested from half of the mice (n = 4 mice per group) in the *S. aureus*-infected and uninfected groups. Bacteria adherent to the implants were detached by sonication in 1 ml 0.3% Tween-80 in TSB for 10 minutes followed by vortexing for 5 minutes [11]. Bacteria in the joint tissue were isolated by homogenizing bone and joint tissue from the infected knee (Pro200® Series homogenizer; Pro Scientific, Oxford, CT). The number of bacterial CFUs obtained from the implant and joint tissue was determined by counting CFUs after overnight culture of plates. For these experiments, the sample size was 4 mice per group.

### Histologic analysis

On day 48, mice were euthanized and joint specimens from half of the mice (n = 4 mice per group) in the *S. aureus*-infected and uninfected groups were fixed in formalin (10%) overnight. Specimens were decalcified by incubation in Decalcifier II® solution (Surgipath Medical Industries, Inc., Richmond, IL) for 6 hours and specimens were processed and embedded in paraffin. Sagittal sections of 4 µm thickness were cut and then were stained with hematoxylin and eosin (H&E) and Gram stain by the UCLA Translational Pathology Core Laboratory (TPCL) according to guidelines for clinical specimens.

### Statistical analysis

Data were compared using a Student's t-test (two-tailed). All data are expressed as mean ± standard error of the mean (sem). Values of p<0.05 were considered statistically significant.

## Results

### *In vivo* optical imaging to monitor the bacterial burden and neutrophil infiltration

To model an orthopaedic implant infection, we used our previously published model which includes the surgical placement of a medical-grade titanium K-wire (Synthes, Inc., West Chester, PA) into the right femurs of mice with the cut end protruding into the joint space [10]–[13]. An inoculum of *S. aureus* strain Xen29 (1×10^3^ CFUs in 2 µl PBS) was pipetted into the joint space before closure. Xen29 possesses the bioluminescent construct in the bacterial chromosome and it is thus maintained in all progeny without selection [29]. Noninvasive and longitudinal monitoring of the *in vivo* bacterial burden and neutrophil infiltration was performed by sequentially measuring the *S. aureus* bioluminescent signals and EGFP-neutrophil fluorescent signals in anesthetized LysEGFP mice on various postoperative days (i.e., days 2, 5, 14, 19, 28, 33 and 48) using the IVIS Spectrum® imaging system (Caliper).

**Figure 1:**
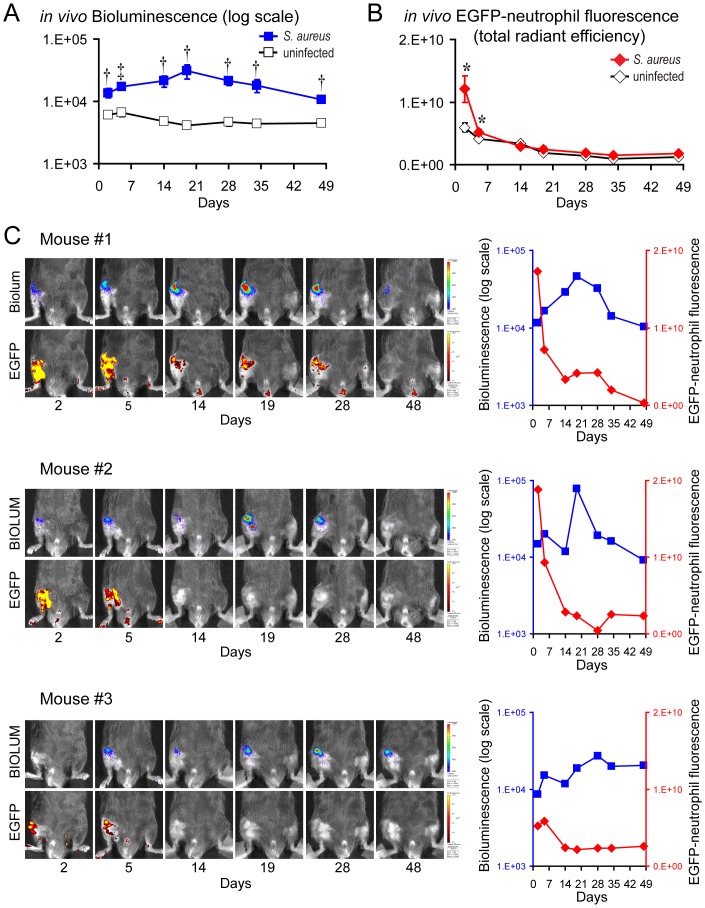
Two-dimensional *in vivo* bioluminescence and fluorescence imaging to monitor the bacterial burden and neutrophil infiltration during an orthopaedic implant infection. *S. aureus* or no bacteria (uninfected) (n = 8 mice per group) were inoculated into the knee joints of LysEGFP mice in the presence of a titanium K-wire implant and mice were imaged using the IVIS Spectrum® imaging system (Caliper). (A) Mean bacterial burden as measured by *in vivo* bioluminescence (mean total flux [photons/s] ± sem) (logarithmic scale). (B) Mean neutrophil infiltration as measured by *in vivo* fluorescence (mean total radiant efficiency [photons/s]/[µW/cm^2^] ± sem). *p<0.05, †p<0.01, ‡p<0.001 *S. aureus*-infected mice versus uninfected mice (Student's t-test [two-tailed]). (C) (Left) Representative images of *in vivo* bioluminescence (upper panels) and *in vivo* fluorescence (bottom panels) overlaid on a grayscale photograph of the mice from the three *S. aureus*-infected mice that had different patterns of the bioluminescent signals. (Right) *In vivo* bioluminescent signals (photons/s/cm^2^/sr) (logarithmic scale) (blue) and *in vivo* fluorescent signals ([photons/s]/[µW/cm^2^]) (red) for each of these three mice.

*S. aureus*-infected mice had bioluminescence signals that peaked on day 19 (3.1×10^4^±8.1×10^3^ photons/s/cm^2^/sr) and were statistically higher (2.3- to 7.5-fold) than the background signals of uninfected mice (∼5×10^3^ photons/s/cm^2^/sr) at all time points through day 48 when the experiment was arbitrarily terminated (Figure 1A). These findings confirm the presence of a chronic *S. aureus* infection at the site of implant infection in the knee joint. The EGFP-neutrophil fluorescent signals of *S. aureus*-infected LysEGFP mice were statistically greater than uninfected mice on days 2 and 5 (1.7-fold and 1.3-fold, respectively [p<0.05]) after which the signals of both infected and uninfected mice similarly approached the background levels for the remainder of the experiment (Figure 1B). This degree of neutrophil recruitment confirms our clinical observations that the inoculum of 1×10^3^ CFUs of Xen29 produced a low-grade inflammatory response, suggesting that EGFP-neutrophil fluorescence provides a quantifiable measurement of the early clinical inflammation observed in this model.

One of the most compelling reasons to use optical imaging technology is to evaluate the specific changes in *S. aureus* bioluminescent and EGFP-neutrophil fluorescent signals in each individual mouse. This is important because there is inherent variability when performing *in vivo* experiments and having the capability to monitor the response in each individual mouse could provide information into the dynamic changes that are occurring longitudinally over time. As an example, data from 3 representative mice were chosen (from the entire group of 8 mice) because these particular mice had the most disparate trends in bioluminescent and fluorescent signals (Figure 1C). The first mouse had a similar pattern as the mean bacterial bioluminescent data for the entire group with the bioluminescent signals peaking on day 19 and remaining above background signals for the entire 48 day experiment. In this mouse, the EGFP-neutrophil fluorescent signals were detectable and greater than the background signals through day 28. The second mouse had a similar trend in bacterial bioluminescent signals that also peaked on day 19, but there was some variability of the bioluminescent signals during the course of the experiment. In this mouse, the EGFP-neutrophil fluorescent signals were detectable on days 2 and 5 but the signals rapidly decreased below the level of detection for the remainder of the experiment. Interestingly, the third mouse had a slightly different pattern with bioluminescent signals that increased during the entire course of the experiment. In this mouse, low levels of EGFP signals were detectable on days 2 to 5 but then they rapidly decreased below the level of detection for the remainder of the experiment. Taken together, this optical imaging technology can be used to provide noninvasive and longitudinal measurements of the bacterial burden and neutrophilic inflammation in each individual mouse over the entire 48 day course of the experiment.

### *In vivo* optical imaging combined with X-ray imaging to monitor the bacterial burden and neutrophil infiltration in two-dimensions

**Figure 2:**
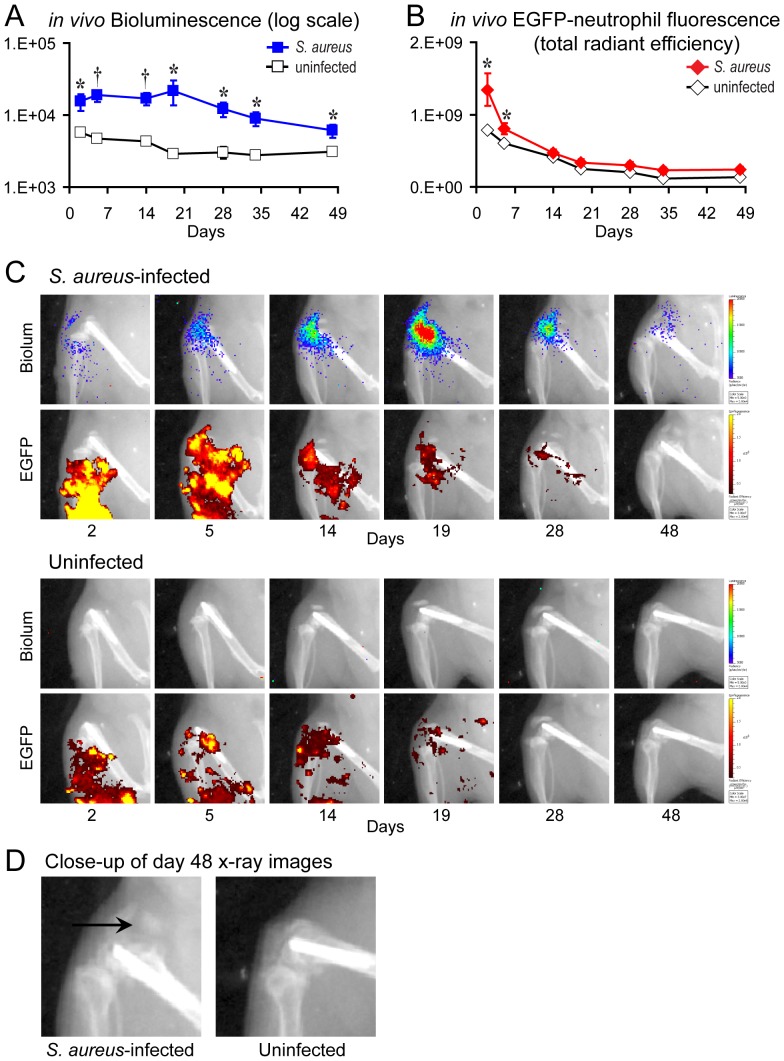
Two-dimensional *in vivo* bioluminescence, fluorescence and X-ray imaging to monitor the bacterial burden and neutrophil infiltration during an orthopaedic implant infection. *S. aureus* or no bacteria (uninfected) (n = 8 mice per group) were inoculated into the knee joints of LysEGFP mice in the presence of a titanium K-wire implant and mice were imaged using the IVIS Lumina XR® imaging system (Caliper). (A) Mean bacterial burden as measured by *in vivo* bioluminescence (mean total flux [photons/s] ± sem) (logarithmic scale). (B) Mean neutrophil infiltration as measured by *in vivo* fluorescence (mean total radiant efficiency [photons/s]/[µW/cm^2^] ± sem). *p<0.05, †p<0.01, ‡p<0.001 *S. aureus*-infected mice versus uninfected mice (Student's t-test [two-tailed]). (C) Representative images of *in vivo* bioluminescence (upper panels) and *in vivo* fluorescence (bottom panels) on a color scale overlaid on an X-ray image of a *S. aureus*-infected mouse and an uninfected mouse. (D) Representative osteolytic lesions (denoted by an arrow) and increased bone size observed on close-ups of the X-ray images at the distal end of the femur at the site of the implant infection in *S. aureus*-infected mice on day 48. These osteolytic lesions were not observed in any of the uninfected mice.

The Lumina XR® imaging system (Caliper) has the capability of detecting optical bioluminescent and fluorescent signals, and overlaying those signals on an X-ray image of the mice. Using this system, we attempted to visualize the location of the *S. aureus* bioluminescent and EGFP-neutrophil fluorescent signals in the context of the anatomical X-ray images of the K-wire implants in the right femurs of the mice (Figure 2). The *S. aureus* bioluminescent signals were visualized at the end of the titanium implants in the right knee joint at the site of bacterial inoculation. In addition, the EGFP-neutrophil fluorescent signals could be visualized in and around the soft tissue at the site of the implant infection in the right knee joint. Thus, the Lumina XR was capable of providing a 2D visualization of the optical signals from the site of implant infection in the knee joints of the mice. Similar to the data obtained using the IVIS Spectrum® (Figure 1), the *S. aureus*-infected mice had bioluminescence signals that peaked on day 19 (2.2×10^4^±8.3×10^3^ photons/s/cm^2^/sr) and were statistically higher (2.7- to 7.5-fold) than the background signals of uninfected mice (∼5×10^3^ photons/s/cm^2^/sr) at all time points through day 48 (Figure 2A, C). The EGFP-neutrophil fluorescent signals of *S. aureus*-infected mice were statistically greater than uninfected mice on days 2 and 5 (1.7-fold and 1.3-fold, respectively [p<0.05]) at which point the signals of both infected and uninfected mice similarly approached the background levels for the remainder of the experiment (Figure 2B, C). Therefore, the bioluminescent and fluorescent signals could be visualized in the anatomical context of the knee joint containing the titanium K-wire implant within the femur as seen on 2D X-ray images. Interestingly, in several of the *S. aureus*-infected mice, osteolytic lesions (denoted by an arrow) and increased bone size were observed on close-ups of the X-ray images at the distal end of the femur at the site of the implant infection (Figure 2D). These lesions were not observed in any of the uninfected mice. Similar osteolytic lesions are typically seen on X-rays of orthopaedic implant infections in humans [19]. However, the limited resolution and inherent limitations of 2D radiography of the X-ray images generated by the Lumina XR® did not permit the degree of osteolysis to be quantified with the preferred degree of accuracy.

### *In vivo* optical imaging combined with μCT imaging to visualize the bacterial burden, neutrophil infiltration and bone damage in three-dimensions

To visualize the bacterial bioluminescent signals and the EGFP-neutrophil fluorescent signals in a 3D anatomical context, we combined the optical IVIS Spectrum® images with μCT images generated by the Quantum FX® *in vivo* μCT system (Caliper). This was made possible because the same mouse imaging shuttle chamber for the IVIS Spectrum® could be placed into the Quantum FX® for sequential optical and μCT imaging to obtain images of each mouse in the exact same orientation. The optical data could then be mapped onto the 3D μCT images using a diffuse optical tomography reconstruction algorithm as previously described [30]. This allowed a 3D reconstruction of the *S. aureus* bioluminescent signals in the infected right knee joint to be co-registered in the anatomical context of the K-wire implant extending into the right knee joint from the femoral intramedullary canal seen on the μCT images. An example of this 3D co-registration from a *S. aureus*-infected mouse on day 5 is shown (see Movie S1).

**Figure 3:**
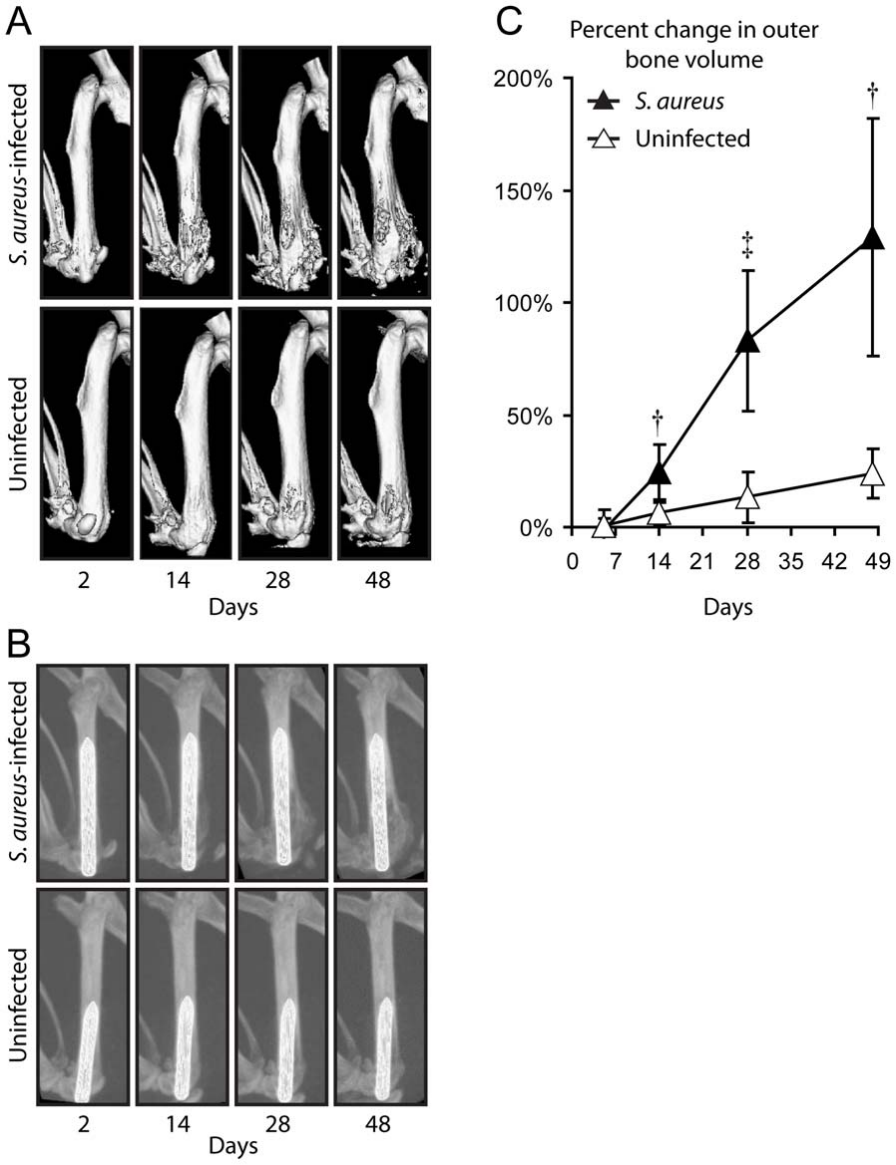
Three-dimensional μCT images to visualize the changes in the bone during an orthopaedic implant infection. *S. aureus* or no bacteria (uninfected) (n = 8 mice per group) were inoculated into the knee joints of LysEGFP mice in the presence of a titanium K-wire implant and mice were imaged using the Quantum FX® *in vivo* μCT system (Caliper). (A) Representative 3D μCT renderings of the femurs implanted with the titanium K-wire implant from *S. aureus*-infected (upper panels) and uninfected mice (lower panels). (B) Representative maximal intensity projection images of the femurs with the titanium K-wire implants taken from a lateral view from *S. aureus*-infected (upper panels) and uninfected mice (lower panels). (C) Percent outer bone volume change of the distal femurs normalized to the initial time point (mean ± sem). †p<0.01, ‡p<0.001 *S. aureus*-infected mice versus uninfected mice (Student's t-test [two-tailed]).

**Figure 4:**
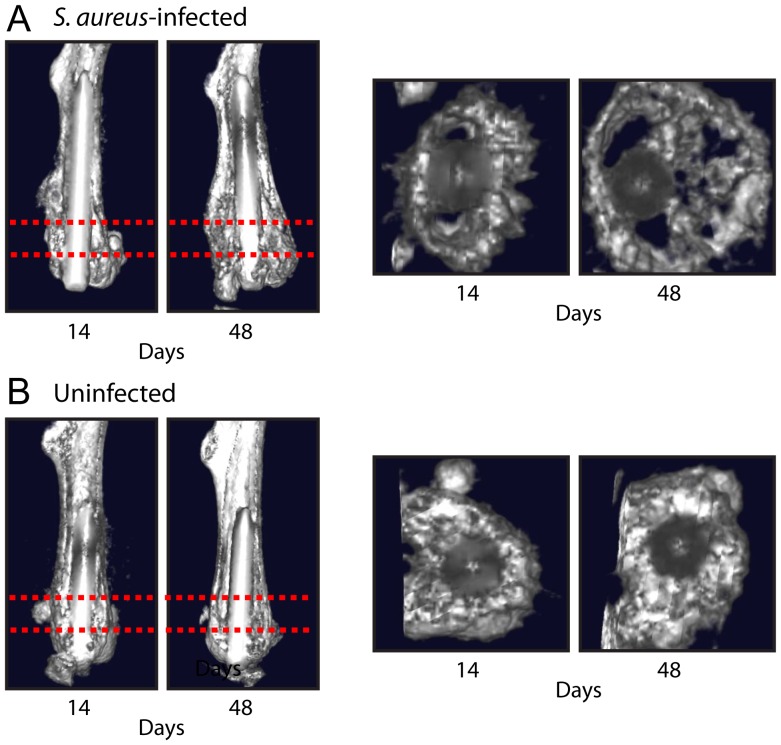
Three-dimensional longitudinal and cross-sectional μCT images to visualize bone changes during an orthopaedic implant infection. *S. aureus* or no bacteria (uninfected) (n = 8 mice per group) were inoculated into the knee joints of LysEGFP mice in the presence of a titanium K-wire implant and mice were imaged using the Quantum FX® *in vivo* μCT system (Caliper). Representative 3D longitudinal (left) and cross-sectional (right) μCT images (from the area within the dashed red lines on the longitudinal images) on days 14 and 48 of femurs implanted with a titanium K-wire implant from *S. aureus*-infected mice (A) and uninfected mice (B).

In addition, μCT imaging provided an opportunity to evaluate the anatomical changes in bone that occurred in response to the implant infection and inflammation. In particular, there was increased outer bone volume of the distal femur observed during the course of infection. This could be seen in 3D renderings (Figure 3A) as well as in maximum intensity projections taken from the lateral view that were scaled to visualize bone around the intramedullary titanium implant (Figure 3B). To quantify the changes in outer bone volume of the femurs, 3D volumetric image analysis was performed on the distal 25% of the femurs. The outer bone volume was found to increase by 24%, 93% and 129% on days 14, 28 and 48, respectively, compared with the initial size measured on day 2 (Figure 3C). The increased distal femur outer bone volume was a consequence of substantial bone damage caused by osteomyelitis and osteolysis, which could be visualized by creating a 3D rendering with a clipping plane (Figure 4). Specifically, a thick cross-sectional section 10% of the femoral length that was positioned near the distal metaphysis (20–30% of the femoral length from the distal end) showed low density signals and bone loss around the implant in *S. aureus*-infected mice (Figure 4A) compared with uninfected mice, which had high density bone surrounding the implant and no evidence of bone loss or damage (Figure 4B). Although image artifacts from the metal pin can affect this rendering, thresholds and clipping planes were kept as consistent as possible to facilitate this comparison.

### Bacterial counts and histologic analysis of post-operative knee joints

**Figure 5:**
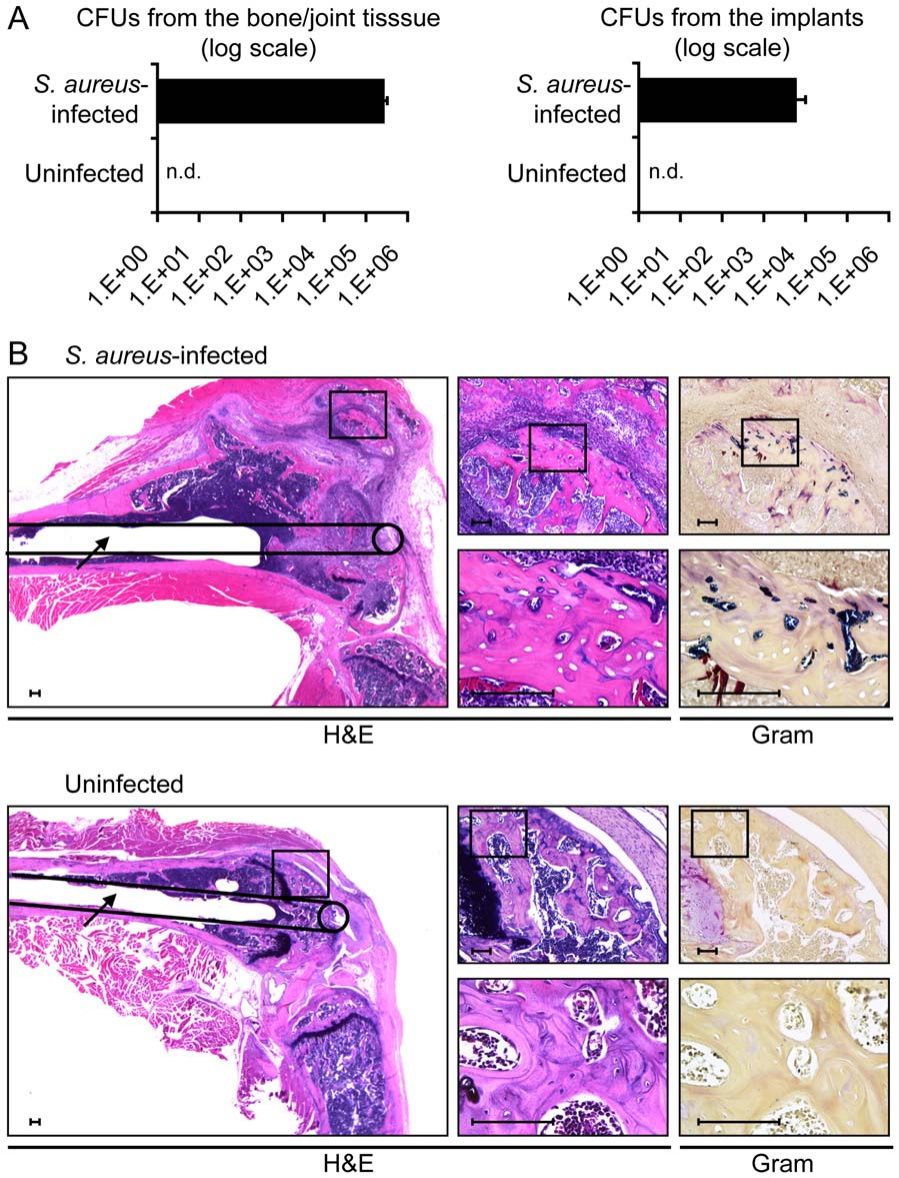
Bacterial Counts and Histologic analysis. *S. aureus* or no bacteria (uninfected) (n = 8 mice per group) were inoculated into the knee joints of LysEGFP mice in the presence of a titanium K-wire implant and mice. At the end of the experiment (on postoperative day 48), the infected and uninfected groups were divided, with half the implants and joint tissue harvested to count the numbers of bacteria and the remainder for histologic analysis. (A) Mean CFUs ± sem (logarithmic scale) isolated from the implants and joint tissue. (B) Representative photomicrographs of histologic sections (1 of 4 mice per group, with similar results). Left large panels: low magnification of hematoxylin and eosin (H&E) stained joint specimens with a line drawing of the location of the implant with the intramedullary canal seen within the femur. Upper right small panels: higher magnification of H&E- and Gram-stained joint specimens of the boxed area in the large left panel at the location of the cut end of the implant within the joint. Lower right small panels: higher magnification of H&E- and Gram-stained joint specimens of the boxed area in the upper right panels. n.d. = not detected. Scale bar = 100 µm.

To confirm that the *in vivo* bioluminescent signals accurately represented the bacterial burden *in vivo*, implants and periprosthetic bone and joint tissue specimens were harvested at the end of the experiment on day 48 and traditional bacterial counts were performed (Figure 5A). CFUs were detected from both the implants and from the bone and joint tissue of all *S. aureus*-infected mice. The *S. aureus*-infected mice had an average of 6.1×10^3^ CFUs harvested from the implants and 2.8×10^5^ CFUs isolated from the bone and joint tissue. As expected, uninfected mice had no bacterial CFUs isolated from either the implants or from the bone and joint tissue. These results confirm that the *in vivo* bioluminescent signals represent a chronic and persistent bacterial infection with bacteria that were present on the surface of the implants as well as from the periprosthetic bone and joint tissue.

To determine the microscopic location of the inflammatory infiltrate and bacterial inoculum within the infected post-operative joints, histologic sections of knee joints were evaluated from *S. aureus*-infected and uninfected mice at the end of the experiment on day 48 (Figure 5B). In hematoxylin & eosin (H&E) stained sections, the *S. aureus*-infected bone had markedly increased size of the distal femur at the site of the implant infection with substantial bone damage. In contrast, the histologic sections from uninfected mice had a normal size of the distal femur and maintained a normal growth plate and cortical bone present around the surgical implant. Furthermore, a marked inflammatory infiltrate was seen in the bone and joint tissue in *S. aureus*-infected mice but not in uninfected mice. Gram-positive bacteria (blue-staining) could be readily detected within the infected-bone in histologic sections from *S. aureus*-infected mice. In contrast, no bacteria were detected by Gram stain in histologic sections from uninfected mice. These histologic findings corroborate our *in vivo* bioluminescence and μCT imaging data, which demonstrated that the *S. aureus*-infected mice had a persistent infection and chronic inflammation, resulting in markedly increased outer bone dimensions and bone damage of the distal femur.

## Discussion

Multimodality imaging that combines optical and structural information provides a new capability to monitor biological processes in a 3D anatomical context [1]–[4]. Since μCT imaging is widely used and provides excellent visualization of bone, the combination of optical and μCT imaging may be particularly useful for studying biological processes that impact bone [26]–[28]. In the present study, we combined optical and anatomical imaging to evaluate the bacterial burden and neutrophilic inflammation in the context of the pathologic changes in the bone in our previously published mouse model of orthopaedic implant infection [10]–[13]. This was accomplished by inoculating a bioluminescent *S. aureus* strain (Xen29 [29]) into the knee joints of LysEGFP mice [25] in the presence of a surgically-placed titanium K-wire implant [10]–[13]. *In vivo* bioluminescent and fluorescent signals were detected using the IVIS Spectrum® imaging system (Caliper) and μCT images of the mice in the same exact anatomical position were obtained by sequential imaging with the Quantum FX® *in vivo* μCT system (Caliper). After reconstruction of the 3D optical signals, this permitted co-registration of the *S. aureus* bioluminescent signals and μCT images using an automated registration routine [30]. Finally, the μCT images were used to evaluate the changes in the bone at the site of the distal femur, which contained the infected K-wire implant. In *S. aureus*-infected mice, there was an increase in outer bone volume with accompanying low density bone signals corresponding to areas of osteomyelitis and osteolysis. In contrast, uninfected mice maintained high density bone surrounding the implants throughout the 48-day duration of the experiment. The validity of these imaging measurements was confirmed with traditional CFU counts, which demonstrated the presence of bacteria on the implants and within the infected bone and joint tissue. Additionally, histologic analysis demonstrated the presence of Gram positive bacteria in bone and joint tissue of *S. aureus*-infected mice as well as greatly increased outer bone dimensions. Taken together, these advanced *in vivo* imaging modalities have provided the capability to investigate the consequences of infection and inflammation on the bone and bone-implant interface longitudinally and noninvasively, thereby expanding the ability of this mouse model to more comprehensively study orthopaedic implant infections.

Since orthopaedic implant infections represent a clinically and economically disastrous complication of orthopaedic surgical procedures [17]–[19], the ability to study both the causes and consequences of these implant infections represent a major technological advancement. Our initial efforts to combine the bioluminescent and fluorescent signals using the IVIS Lumina XR® imaging system (Caliper) allowed visualization and measurements of these optical signals in the context of 2D X-ray images of the bone. However, the limited resolution and limitations of 2D X-ray images did not permit the bone damage to be accurately visualized and measured. We thus attempted to combine the optical signals obtained using the IVIS Spectrum® imaging system (Caliper) followed by sequential μCT images generated using the Quantum FX® *in vivo* μCT system (Caliper). This strategy permitted a more accurate and 3D depiction of the bone damage that occurred during the implant infection. Thus, this multimodality imaging strategy provided noninvasive and longitudinal monitoring of various endpoints (all of which are hallmarks of implant infections in humans), including: 1) *in vivo* bacterial burden, 2) neutrophilic inflammatory response and 3) bone damage [17]–[19].

This mouse model of orthopaedic implant infection combined with these imaging modalities could also be employed to evaluate the ability of bacterial-specific tracers or probes to identify the presence of an infection. This area of investigation has significant translational potential since the diagnosis of an orthopaedic implant infection represents a major clinical challenge. This is because orthopaedic implant infections frequently have a subtle presentation such as pain alone or pain associated with implant loosening, which could be caused by nonspecific inflammation or aseptic loosening rather than an infection. The laboratory tests and imaging studies currently used to help diagnose an implant infection include determining the levels of ESR and CRP in the blood, evaluating the numbers of white blood cells (especially neutrophils) in the synovial fluid, performing bacterial cultures of synovial fluid, joint tissue or bone and radiographic and other imaging studies [17], [18]. However, these tests do not accurately distinguish between inflammation and infection, especially since bacterial cultures are often negative even in the presence of an infection [17], [18]. Previous studies have reported that bacterial-specific fluorescent probes could be used to detect the presence of a bacterial infection *in vivo*. One study found that maltodextrin-based imaging probes (MDPs), which are composed of a fluorescent dye conjugated to maltohexaose and are internalized by bacteria via a bacterial-specific maltodextrin transport pathway, can be used in combination with similar noninvasive fluorescent *in vivo* imaging to detect bacteria during a thigh infection *in vivo* [31]. Another study found that fluorescent-labeled prothrombin derivatives that bind to staphylocoagulase in combination with fluorescence molecular tomography fused to X-ray computed tomography (FMT-CT) could be used to detect the presence of a *S. aureus* infection in endocarditic vegetations [32]. Our model provides an opportunity to evaluate whether these or other bacterial-specific tracers and probes could identify the presence of the infection/biofilms on the metal implants, which may lead to the development of new and accurate diagnostic methods to detect the presence of an orthopaedic implant infection in humans.

It is important to note that this entire study was completed using only 16 mice (8 mice in the *S. aureus* infected group and 8 mice in the uninfected control group). This low animal usage underscores one of the major advantages of multimodality *in vivo* imaging, which is to provide longitudinal measurements of different endpoints (e.g., bacterial burden, neutrophilic inflammation, and bone damage) in the same animals [11], [13]. Other methods to determine these endpoints require much larger animal usage because euthanasia is required to obtain data for each endpoint (e.g., traditional CFU counting or histologic analysis) and at every single time point. Thus, multimodality *in vivo* imaging represents a noninvasive and cost-effective method to obtain longitudinal measurements, which is important when considering the reduction, refinement and replacement of animals used in research and testing. In addition, the measurements obtained for each individual mouse could also be tracked. This is best demonstrated in Figure 1C in which mouse #3 had a slightly different pattern of bioluminescent signals, which steadily increased during the course of the infection. Mouse #3 also had less neutrophil infiltration at early time points as measured by EGFP-neutrophil fluorescent signals. This response (and potentially any other responses that differ among the individual animals) could be the focus of future experiments to determine the host response or bacterial mechanisms that contributed to these responses.

There are some limitations with this mouse model and the *in vivo* imaging performed in this study. First, we recognize that this model oversimplifies the actual procedures performed in orthopaedic surgery in humans. For example, in total knee arthroplasty, the cartilage is removed, and an implant is placed on both the tibial and femoral side and a variety of implant materials in addition to titanium are used (e.g., cobalt-chrome and polyethylene plastic) [33]. However, we believe that this model does reproduce the *in vivo* behavior of orthopaedic implant infections, which are characterized by the chronic and persistent bacterial infection of the implant and the surrounding bone and joint tissue [17]–[19]. Second, the ability to obtain high resolution of the bone in the μCT images was limited because acquisition protocols were designed for low doses of X-ray radiation to prevent any adverse affects on animal health. This is important because higher radiation doses have been shown to have detrimental effects on bone morphology [34] and lower doses, which are comparable to the doses used in this study, have little or no impact on biological processes in bone [35], [36]. However, if longitudinal imaging is not required, higher doses of radiation for μCT imaging can be used on bones harvested *ex vivo* (after mice have been euthanized) to obtain better quality 3D reconstructed bone images [37]. In addition, metal artifacts arising during μCT imaging could interfere with visualization of the bone-implant interface and euthanizing mice and removing the implants can eliminate these artifacts [38]. However, removing the implant for an *ex vivo* terminal imaging study eliminates the ability to monitor the dynamic changes occurring longitudinally over time. Furthermore, it is difficult to physically remove the implant while preserving the bone-implant interface as bone and tissue remain adherent to the implant. Therefore, to minimize metal artifacts, we used 0.8 mm titanium K-wires in this longitudinal study because they caused far fewer metal artifacts than observed with 0.6 mm stainless steel K-wires (data not shown). The impact of any residual image artifacts on the measured data were minimized by a using a semi-automated contouring approach for volume and by excluding data directly dependent on grayscale values. It should also be mentioned that the length of the K-wire implants within the femurs ranged from 7 to 10 mm in length due to differences in how much of the K-wire implant could easily be inserted without force as our prior experience determined that forceful insertion sometimes resulted in a fracture that would confound our results. This explains why the K-wires in the representative images have different lengths. However, the range in length of the implants was similar between the infected and uninfected groups. Since osteolytic lesions were only seen in the infected mice, the presence of the infection rather than the length of the implant was the major determinant in inducing osteolysis.

In summary, multimodality imaging that combines bioluminescence and fluorescence optical imaging with X-ray and μCT imaging has improved this mouse model of an orthopaedic implant infection. This study demonstrates the feasibility of monitoring the ongoing infection and inflammation as well as the impact of these processes on the bone and the bone-implant interface. This model provides a noninvasive preclinical approach to study the pathogenesis of bone damage and osteolysis during this orthopaedic implant infection. Finally, this mouse model and the imaging modalities presented could be used to evaluate potential antimicrobial, anti-inflammatory and therapeutic interventions that could be specifically designed to protect the bone and bone-implant interface as well as to develop new methods to help accurately detect the presence of an orthopaedic implant infection.

## Supporting Information

Movie S1 A three-dimensional μCT image rotated on the vertical axis of the *S. aureus* bioluminescent signals in an infected right knee joint on day 5 in the anatomical context of the K-wire implant extending into the right knee joint from the femoral intramedullary canal.
(MPG)
